# Evaluating China’s mental health policy on local-level promotion and implementation: a case study of Liuyang Municipality

**DOI:** 10.1186/s12889-018-6315-7

**Published:** 2019-01-07

**Authors:** Wei Zhou, Yu Yu, Xinyi Zhao, Shuiyuan Xiao, Lizhang Chen

**Affiliations:** 1 Department of Epidemiology and Health Statistics, School of Public Health, Central South University, 110 Xiangya Road, Changsha, 410078 China; 2 Hospital Administration Institute, Xinagya Hospital, Central South University, 87 Xiangya Road, Changsha, 410078 China; 3 Evaluation Department, Xiangya Hospital, Central South University, Xiangya Road, Changsha, 410078 China; 4 Institute of Medical Humanities, Peking University, No.38 Xueyuan Road, Beijing, 100191 China; 5 Department of Social Medicine and Health Management, School of Public Health, Central South University, 110 Xiangya Road, Changsha, 410078 China; 6 Department of Epidemiology and Health Statistics, School of Public Health, Central South University, 110 Xiangya Road, Changsha, 410078 China

**Keywords:** Mental health policy, Local promotion and implementation, Evaluation, China

## Abstract

**Background:**

Since 2000, a series of national policies have been released to tackle mental health problems in China; however, their promotion and implementation at local level are under-evaluated. This study will evaluate the case of Liuyang Municipality, to present a deeper understanding of China’s problems and lessons on implementation of mental health policy in developing countries.

**Methods:**

A rapid appraisal was conducted on Liuyang’s mental health policy and plan, under two evaluation frameworks: (1) the WHO checklists for mental health policy and plan, (2) activities and time frames stated in Liuyang’s mental health policy and plan. Documentation review, semi-structured interviews with nine key informants, and surveys on 32 front-line implementers were performed. Descriptive statistics and framework analysis were employed respectively to analyze quantitative and qualitative data.

**Results:**

As a local-level promotion of national mental health policies, Liuyang’s mental health policy and plan had evidence base and received highest-level approval within local health system, but without stakeholders’ consultation. The vision, principles and objectives were consistent with national policies. Twelve WHO-suggested areas for actions were unequally covered. Policy content had operational defects of lacking necessary details on evaluation, funding and activity. For implementation, health departments generally outperformed non-health ones. Discrepancies between planning and practices, and uneven regional implementation compromised implementation quality. Insufficient and poorly trained human resources, unguaranteed funding and low client acceptability were identified as implementation barriers.

**Conclusions:**

The case of Liuyang is an active attempt to promote and implement national mental health policies at local level. As a reflection of mental health policy in China and developing countries, its highlights and problems demonstrate that evidence base, high-level approval, multi-sector involvement, operational content, and clear focuses promote policy implementation.

**Electronic supplementary material:**

The online version of this article (10.1186/s12889-018-6315-7) contains supplementary material, which is available to authorized users.

## Background

Mental health has become a public health concern in China. A recent epidemiological study reports that the adjusted 1-month prevalence of any mental disorder among the adult population during 2001–05 is 17.5% [1]. Disease burden studies demonstrate that mental and behavioral disorders account for 23.6% of China’s years lived with disability in 2010 [2].

In response, the central government has put growing priority on mental health since 2000 and a number of mental health policies have been released, including the National Mental Health Plan (2002–2010, 2015–2020) [3, 4], the Proposal on Further Strengthening Mental Health Work [5], the Guidelines for the Development of National Mental Health System (2008–2015) [6], and a series of policies on psychosis management and intervention originated from the National Continuing Management and Intervention Program for Psychosis (NCMIPP) [7–9]. Priorities of these policies include: (1) establishing a government-led and multi-sector-participated mechanism for mental health work, management and coordination; (2) establishing and strengthening mental healthcare system, with emphasis on prevention, rehabilitation, community services and through capability building of professionals and organizations; (3) establishing and implementing mental health legislation for patients’ rights protection; (4) establishing and strengthening psychosis management and interventions; (5) identifying priority population for interventions, like children and adolescents, women and the elderly; (6) increasing the public’s awareness and knowledge of mental health; (7) strengthening research and surveillance on mental health.

Though national policies provide an overall guidelines and set some targets and evaluation indicators for mental health work, local-level promotion and implementation remain essential for exerting policy influence. Therefore, formulating and implementing local mental health plans is proposed in China’s National Mental Health Plan (2002–2010) [3] and it has been further stipulated in China’s first national Mental Health Law (Article 60) [10]. So far, local governments, like ones in Zhejiang Province, Guangdong Province, Qingdao Municipality and Yinchuan Municipality, have published their regional mental health policies [11–14].

Studies in countries, like Sweden, Lao and South Africa, reveal that national health policies’ promotion and implementation at local level commonly encounters problems, including incoherence in policy content and governance, and no or partial implementation [15–17]. However, literature on China’s mental health policy at local level is limited. There is a policy content assessment on six local mental health laws in Mainland China, which identifies problems of unclear regulations and under-emphasized key areas like involuntary hospitalization and protection of vulnerable groups [18]. The other two include a WHO-AIMS (World Health Organization-Assessment Instrument for Mental Health Systems) based evaluation on mental health system in Hunan Province with brief information of local mental health policies [19], and a descriptive report on Liuyang mental health policy and related work [20].

This study will address a gap in China’s mental health policy literature with respect to local-level promotion and implementation. It will present a deeper understanding on China’s problems and general lessons for implementing mental health policy at local level, through answering two key research questions: (1) how is the quality of China’s mental health policies’ promotion and implementation at local level? (2) what are the barriers, specific to policy formulation, content and implementation? This study chooses the case of Liuyang Municipality and for the first time attempts to evaluate one specific municipal mental health policy and its complementary plan on formulation process, policy content, implementation progress and barriers.

## Methods

### The study location and policy brief

Liuyang Municipality is a county-level city under the jurisdiction of Changsha Municipality, the capital city of Hunan Province. By 2015, Liuyang had a population of 1.47 million across 4 urban districts and 28 rural towns, with 99.79% Han ethnicity. It had gross domestic product (GDP) of 16.14 billion U.S. dollars [21], with a higher per capita GDP than the average level of Hunan Province and China at the same year (Table 1) [22, 23]. Medical insurance coverage rate in Liuyang was 95.57% [21]. Liuyang Psychiatry Hospital (PH) is the only local mental health hospital and it is also responsible for the operation of Liuyang Mental Health Prevention and Treatment Center (MHC). It had over 500 beds, 26 psychiatrists and 40 nurses by 2015. As one of the earliest 60 national demonstration sites, Liuyang has participated in NCMIPP since 2005. Liuyang has developed a tiered mental health system, extending from Liuyang MHC to township hospitals to village clinics. Liuyang mental healthcare system provides not only clinical services, but also screening and case management for patients with psychosis (PWP) at communities and advocacy among general population [20].

**Table 1:** Liuyang’s economic development and mental health resources in comparison with Hunan Province and China, 2015

Region	Per capita GDP (US dollars)	Mental health resources per 100,000 population		
		Psychiatrists	Nurses	Beds
Liuyang Municipality	10,979.6 [21]	1.77*	2.72*	34.03*
Hunan Province	7043.9 [22]	2.15**	5.32**	44.99**
China	8090.3 [23]	1.99 [24]	4.16 [24]	24.68 [24]

*Unpublished data from Liuyang MHC;

**Unpublished data from Hunan MHC

Liuyang is the first municipality in Hunan Province to release municipal mental health policy and plan, entitled “the Mid- and Long-term Development Plan for Mental Health in Liuyang Municipality” and “the Plan of Mental Health Work in Liuyang Municipality” (hereinafter referred to as Liuyang Policy and Liuyang Plan respectively). The two documents (translated full texts in Additional file 1: Appendixs 1, 2) have provided guidance for local mental health development from 2007 to 2017 [20]. Four main objectives of Liuyang Policy and Liuyang Plan include: (i) establishing leadership and coordination mechanism for mental health work, (ii) constructing a three-level network of mental health services, (iii) PWP management and intervention, and (iv) improving of the public’s awareness and knowledge of mental health.

### Evaluation frameworks

(1) WHO checklists for mental health policy and plan: as part of WHO Mental Health Policy and Service Guidance Package, the checklists are developed to help draft and evaluate mental health policies and plans [25]. The checklists provide items to examine whether formulation follows key processes (Process Issues), whether content has structure and essential issues (Content Issues), and whether plan is operational considering time frames, targets, evaluation indicators, and funding (Operational Issues) (Table 2) [25]. The item selection is based on literature review, consultation with policy makers, planners and health professionals, and gives full consideration to best practices from low, middle, and high income countries [26].

**Table 2:** Summary of the WHO checklists for mental health policy and plan

Evaluation Section	Examining areas	Brief description
**Checklist for Mental Health Policy**		
Process Issues	Mandate & approval	• The level of mandating to develop & approving policy;
	Evidence base	• Situation & needs assessment; • Other country experience; • Research;
	Consultation	• Consultation with stakeholders;
Content Issues	Vision, principles & objectives	• Realistic & clear statement; • Promoting key values; • Internal consistency;
	Action areas & key groups	• Suggested action areas in clear statement & in a committed way; • Consideration for key groups; • Consistent with other related policies;
**Checklist for Mental Health Plan**		
Process Issues	Mandate & approval	• The level of mandating to develop & approving policy;
	Evidence base	• Situation & needs assessment; • Other country experience; • Experience from other related policy/documents;
	Consultation	• Consultation with stakeholders;
Operational Issues	Strategy	• Strategy for priority action areas;
	Time frames, indicator & targets	• Availability, feasibility and suitability;
	Activities	• Clear defined; • Responsibility, time, outputs, obstacles & funding;
Content Issues	Action areas	• Suggested action areas; • Integrated into other related plans.

Compared with the other related evaluation tool, WHO-AIMS [27], the checklists are the only one designed specifically and exclusively for mental health policy and plan, covering policy formulation and content. The checklist for policy has been employed to assess mental health policies in Ghana, South Africa, Uganda and Zambia [17, 28–30]. It will guide evaluation Liuyang Policy and Liuyang Plan’s quality of policy formulation and content.

(2) Activities and time frames in Liuyang Policy and Liuyang Plan: policy implementation progress will be examined according to regulations on activities and time frames in Liuyang Policy and Liuyang Plan (Table 3).

**Table 3:** Summary of Liuyang Policy & Liuyang Plan’s activities & time frames

Evaluation section	Examining areas	Brief description
Implementation issues	Policy activities	• Leadership and coordination mechanism for mental health; • Mental health service network construction; • PWP surveillance, management & interventions; • Interventions for other key population; • Dissemination & popularization of mental health knowledge; • Financing.
	Evaluation indicators	• Mental health literacy rate among the general population (by 2010 & 2017); • Prevalence of mental disorders among children & adolescents (by 2010); • Training rate of mental health professionals (by 2010); • Detection rate of psychiatric illnesses (by 2015); • Treatment rate for schizophrenia (by 2010 & 2015); • Rate of PWP’s crimes & violent/disruptive behaviors (by 2010 & 2015); • Rate of PWP under guardianship (by 2010 & 2015); • Rate of PWP’s significant improvement (by 2015); • Coverage rate of mental health prevention, treatment& rehabilitation work (by 2010).

### Data collection

A rapid appraisal was conducted from March to May of 2015. Rapid appraisal is “an approach for quickly developing a preliminary understanding of a situation where specific research techniques are chosen from a wide range of options” [31]. Data triangulation is the core of its validity and defensibility [31–33]. This approach works best in homogeneous communities and has been widely applied to researches on rural development, nutrition, primary healthcare and process evaluation on strategies [31–34]. Considering Liuyang’s homogeneity in the aspects of population’s demographic characteristics (e.g. ethnicity and urban-rural distribution) and social welfare (e.g. medical insurance coverage), rapid appraisal will be an appropriate method for this case. In this study, documentation review, semi-structured interview and survey were selected for data collection. Information obtained by WZ and YY through multiple methods from different sources was cross-checked for reliability.

(1) Documentation review: Mental-health-related national policies, plans, laws and regulations from 2002 to 2015 (Additional file 1: Appendix 3) were reviewed to examine Liuyang Policy and Liuyang Plan’s consistency with them. Secondary data (Additional file 1: Appendix 3), including Liuyang government reports covering mental health issues, research on Liuyang mental health and routine surveillance data from Liuyang MHC, were retrieved to provide evidence for policy implementation progress.

(2) Semi-structured interview: According to the evaluation frameworks, interview questions were designed to cover policy formulation, implementation progress and barriers (Additional file 1: Appendix 4). Major policy formulators of Liuyang Policy and Liuyang Plan, who were also major policy implementers at senior and middle levels, were all interviewed (Table 4). One randomly-selected mental health officer (MHO), as a front-line-level implementer, was interviewed. Two patient households receiving PWP management and interventions, as policy recipients, were selected according to patients’ social function levels. The information provided by the above interviewees satisfied the principle of data saturation [35] for the purpose of data triangulation. All interviews were conducted face-to-face or telephonically in Mandarin Chinese, lasting for 15 minutes to 1 hour each. Interview outlines were tailored to respondents’ roles. With oral informed consents, four interviews were digitally recorded and taken written notes, but one recording damaged during transcribing; the other five refused to be recorded but agreed to be taken notes.

**Table 4:** Samples for semi-structured interviews

Interviewees	n	Role	Mean interview time
Senior leaders of Liuyang MHC&PH	3	Policy formulators & implementers	45 min*
Department directors of Liuyang MHC&PH	2	Policy formulators & implementers	40 min
MHOfrom a town hospital**	1	Policy implementers	25 min
PWP ***	1	Policy recipients	20 min
Family members of patients	2	Policy recipients	20 min

*One interview lasting for less than 30 min;

**The MHO had two year work experience of PWP management and interventions;

***One of the two interviewed households had four patients with schizophrenia, who all had cognition impairment and were excluded for interview

(3) Survey: there were 37 town hospitals or community healthcare centers in Liuyang Municipality and each had one MHO. At the annual training for MHOs, an independent and self-reported survey (Additional file 1: Appendix 5) was undertaken among the 37 MHOs on voluntary and anonymous principle and 32 valid questionnaires were received. The survey was designed based on activities and time frames stated in Liuyang Policy and Liuyang Plan and used to cross-check the implementation progress. Perceived barriers were questioned open-ended.

### Data analysis

All data in Chinese were not translated into English, to avoid loss of meaning. Data relevant to questions in the evaluation frameworks were first picked out by two bilingual researchers (WZ & YY) and then categorized into formulation process, content, implementation and barriers for further analysis.

For qualitative data like full texts of Liuyang Policy and Liuyang Plan, interview transcripts and answers to open-ended survey questions, framework analysis was performed, using Excel spreadsheets [36, 37]. Based on the study objectives and the adopted evaluation frameworks, an analytical framework consisting of themes was decided by four researchers (WZ, YY, LZC & SYX) through a process of iteration. After that, two researchers independently conducted coding under the analytical framework, with additional themes added to the analytical framework as determined by the data. Disagreement was resolved through consensus or a third researcher’s (SYX) opinions. Quantitative data, obtained from secondary data or survey, were either used directly or after descriptive statistics, to answer evaluation questions.

## Results

### Formulation process issues

#### Mandate, level of approval and official dissemination

Liuyang PH firstly proposed to formulate a local mental health policy and directed policy drafting in 2006, the next year after Liuyang participated in NCMIPP. The formulation had a clear purpose of promoting NCMIPP related work. In 2007, Liuyang Municipal Health Bureau “semi-formally” approved Liuyang Policy and Liuyang Plan.

“*In 2007, our hospital submitted the policy (draft) to the Municipal Health Bureau for approval…Liuyang (Municipal Health Bureau) has approved 24 positions for public health staff…” (A, Policy formulator and implementer).*

*“This policy has been approved by the Municipal Health Bureau, and Liuyang (government) has included it in the general health plan.” (Interviewer: But I cannot find the two documents (referring to Liuyang Policy and Liuyang Plan) on the government website, so only the staffing positions of public health or the whole policy got approved?)“There may be no formal documents. However, after our hospital submitted the policy, the Municipal Health Bureau officially responded that they agreed for us to conduct work according to the policy. Because this policy also involves town hospitals for issues like appointing MHOs, agreement from Municipal Health Bureau is needed.”(B, Policy formulator and implementer).*

The Twelfth Five-Year Plan of Liuyang Regional Health Development (2011–2015) included part of the policy content, like the construction of mental healthcare network and some evaluation indicators of PWP management and interventions [38].

#### Evidence base and consultation

According to interviews with policy formulators, national mental health policies, especially ones related to NCMIPP, provided important guidance in the drafting of Liuyang Policy and Liuyang Plan. In addition, a university research team, with expertise of mental health and public health, was employed to provide technical support for the policy formulation. Under the team’s suggestions, a sampling survey was conducted among local residents for prevalence of mental disorders, services utilization and mental health literacy [39, 40]. However, consultations with stakeholders, like non-health departments, patients and their families, were missing.

### Content issues

#### Vision, principles and objectives

The overall structures of Liuyang Policy and Liuyang Plan were complete, including current situation, guiding principles, goals/objectives, organization and management, and measures. Vision was not written as a separate section, but was described at the beginning as *“… (to) guarantee the benefits of mental health patients, improve the mental health level of the general population, maintain the stability of our city, reduce cases of ‘entering and re-entering poverty because of diseases’, and establish a harmonious society…”*. Guiding principles of both documents intensely reflected Chinese leaders’ governance philosophy and national mental health work requirements. Most values and principles suggested in the WHO checklists, including human rights, social inclusion, community care, integration, evidence-based practice and intersectoral collaboration, were also covered and scattered throughout the two documents.

Four main objectives were clearly stated in Liuyang Policy. Based on local situation in Liuyang, the four were selective responses to national mental health priorities, including the inter-ministerial joint conference system for mental health work [41], nationwide mental health system development [3, 5], NCMIPP [7], and increasing the public’s awareness and knowledge [3, 5].

#### Action areas and operationality

Strategies to achieve the four policy objectives unevenly covered action areas suggested in the WHO checklists. First, two action areas, including “quality improvement” and “procurement & distribution of essential medicines” were not covered. Second, five action areas were somewhat emphasized, but with obvious problems. For “financing”, a specific budget for Liuyang MHC’s operation was calculated, but no details on budget for the whole mental health system or any specific activity. Measures for “legislation & human rights” were limited and weak, only claiming “solving the issue of locking up PWP” and “accelerating the mental health legislation process”. The development and operation of “information systems” were confined to psychosis surveillance. For “human resources & training”, staff quotas within the mental healthcare network were defined and training was required to be provided by Liuyang MHC; however, issues like qualification, recruitment, retention, assessment were un-tackled. “Research” was not covered and “evaluation” set indicators mainly for PWP management and interventions. Third, five action areas were strongly emphasized. There are institutional arrangements for “coordination & management”, including a multi-department leadership team, a coordination mechanism, and Liuyang MHC. For “organization of services”, constructing a village-town-city-level network of mental healthcare with an emphasis on community approach was defined in details. Integrating mental health services into general hospitals and maternal & children’s hospitals was also required. Interventions for children and adolescents, women, and the elderly, like early screening and adaptability training, were adopted for “promotion and prevention” and assumed by Liuyang MHC, schools, Women’s and Elders’ Federations, and maternal & children’ hospitals. “Rehabilitation” work mainly relied on Liuyang PH. “Advocacy” was repeatedly emphasized and involved health professionals, teachers, social societies and media. “Intersecotral & intrasectoral collaboration” was intensely reflected in the multi-department leadership team, multi-party-involved promotion, prevention and advocacy, and service integration.

Considering policy operationality, targets, time frames and evaluation indicators were consistent with national ones, but mainly set for priority strategies, like PWP management and interventions (Table 5). Activities and responsibility for different organizations were stated (Table 6), but not detailed enough for accurate implementation. For example, MHOs’ routine visit to PWP was not defined in frequencies, methods and coverage rate. Training local mental health professionals by Liuyang MHC was not defined in frequencies either. Funding for most activities was not mentioned, and there were no calculation on costs and prediction for obstacles.

**Table 5:** Targets, baseline and progress of Liuyang Policy & Liuyang Plan, in comparison with national requirements

Evaluation indicators	Liuyang requirements	National requirements		Liuyang Baseline (2007)	Liuyang Progress (Mar. 2015)
		Set before 2007	Set after 2007 (latest by 2015)		
Mental health literacy rate among the general population	50% (by 2010)	30% (by 2005) [3]	50% (by 2010) [3, 6]	< 30%*	58.3% among rural adults by 2011 [42]
	80% (by 2017)	50% (by 2010) [3]	80% (by 2017) [6]		
Prevalence of mental disorders among children & adolescents	lower to 12% (by 2010)	lower to 12% (by 2010) [3]	lower to 12% (by 2010) [3]	Unknown	Unknown
Training rate of mental health professionals	80% (by 2010)	50% (by 2005) [3]	80% (by 2010) [3]	21.8% [43]	100% **, ***
		80% (by 2010) [3]			
Detection rate of psychiatric illnesses	over 0.8% (by 2015)	–	0.4% for psychosis (by 2015) [43]	0.4% for psychosis**	0.38% for psychosis ***
Treatment rate for schizophrenia	60% (by 2010)	30% (by 2005)	80% (by 2015) [6]	< 30% for psychosis*	73.18% for psychosis ***
	over 60% (by 2015)	60% (by 2010) [3]	> 80% (by 2020) [4]		
Rate of PWP’s crimes & violent/disruptive behaviors	< 0.2% (by 2010)	–	–	Several incidents, without specific number**	1 incident by PWP in last year ***
	almost eradication (by 2015)				
Rate of PWP under guardianship	90% (by 2010)	–	–	Unknown	98.93% for PWP ***
	> 95% (by 2015)				
Rate of PWP’s significant improvement	> 70% (by 2015)	–	60% for registered PWP in stable condition (by 2012) [44]	Unknown	75.82% for registered PWP in stable condition ***
Coverage rate of mental health prevention, treatment& rehabilitation work	75% (by 2010)	covering a population of 400 million by 2005 & 800 million by 2010 [3]	covering a population of 400 million by 2005 & 800 million by 2010 [3]	Unknown	Unknown

* Data from the sampling survey for Liuyang policy formulation **Data provided by Liuyang MHC *** Results of survey among MHOs

**Table 6:** Implementation status of policy activities in Liuyang Policy & Liuyang Plan

Implementers	Policy activities	Implementation status
Municipal government	• The leadership team for mental health;	+
	• Working mechanism of multi-department coordination;	+
	• Financing for mental health;	+
	• Medical aid and relief to PWP;	+
Liuyang MHC/PH	• Mental health education;	+
	• Mental health professionals’ training;	+
	• The information system of PWP;	+
	• Regular guidance for treatment scheme and risk assessment of PWP;	+
	• Evaluation on Liuyang mental health work;	+
	• Medical treatment and rehabilitation;	+
	• Liuyang mental health information website;	0
Town hospitals	• Mental health education;	+
	• PWP screening, reporting, visit and management;	+
Village/community health centers/village committees	• Reporting potential PWP;	+
	• PWP management;	+
General hospitals, maternal &children hospitals	• Mental health education;	0
	• Early screening & psychological consultation;	0
Education department (primary & middle schools)	• Inclusion of mental health education into curriculum;	0
	• Early screening & psychological consultations;	0
	• Mental health training for school teachers and doctors;	0
Women’s & Elders’ Federations	• Mental health education;	0
	• Early screening.	0

0 Not at all implemented + Implemented

### Implementation issues

#### Implementation progress and quality

When being evaluated, Liuyang Policy and Liuyang Plan were at the ninth year of implementation. Interviews and survey results demonstrated that health department, especially the three-level mental healthcare network, implemented most of their policy activities; however, non-health departments, like the education system, did not carry out the policy (Table 6). Because of top-down supervision and financial guarantee from the central government [44–46], targets on PWP management and interventions were in very good progress (Table 5). Meanwhile, being a local priority, PWP management and interventions in Liuyang even outperformed the national average level in several indicators, like treatment rate and proportion of detected and registered psychosis in stable condition [47].

When taking a closer examination, implementation quality had problems. On the one hand, there were discrepancies between planning and practice. For example, the actually allocated funding for Liuyang MHC had an annual gap of 240,000 RMB for personnel and 300,000 RMB for operation. Progress of Liuyang Policy and Plan was evaluated by indicators partially different from originally set ones, like rate of PWP significant improvement replaced by rate of registered PWP in stable condition (Table 5). Meanwhile, the leadership team for mental health was supposed to be led by municipal government, responsible for supervising all mental health tasks in Liuyang. However, in practice, the team was led by local Political and Legal Affairs Commission and mainly focused on management of PWP’s violent or socially disruptive behaviors.

*“We have a team similar to the leadership team for mental health; however, its purpose is to manage (patients’) violent or socially disruptive behaviors. The team is led by the Political and Legal Affairs Commission, and other departments like Health, Civil Affairs, Public Security, Justice and Federation of Disabled Persons will attend. One to two meetings will be organized annually. As a main implementing organization in health sector, our hospital will also attend the team meetings.” (A, Policy formulator and implementer).*

On the other hand, implementation varied among different regions. One typical example was the routine visit to PWP, in terms of for frequencies, methods and coverage (Table 7). For another example, mental health training for village doctors was mandated to town hospitals, and the survey results demonstrated that the number of last-year training varied in towns (Table 7).

**Table 7:** Main results of survey among MHOs

Characteristics of MHOs *n* = 32			
Age, Mean (Std)			38.5 (8.7)
Male, n(%)			20 (62.5)
Major of higher education, n(%)			
Clinical medicine			16 (50)
Nursing			8 (25)
Public Health			4 (12.5)
Pharmacy			4 (12.5)
Professional qualification, n(%)			
Physician (Assistant) License			14 (43.8)
Nurse License			8 (25)
Public Health Practitioner (Assistant) License			3 (9.4)
Pharmacist License			4 (12.5)
Without medical license			3 (9.4)
Part-time, n(%)			32 (100)
Being Mental Health Officer for over 3 years*, n(%)			9 (28.1)
Times of in-service training per person-year, Mean (Std)			0.8 (0.5)
Mental health work of towns n = 32			
Detection rate of PWP (%), Mean (Std)			0.38 (0.6)
Treatment rate of PWP (%), Mean (Std)			73.18 (25.1)
Rate of PWP in stable condition (%), Mean (Std)			75.82 (34.7)
Rate of PWP under guardianship (%), Mean (Std)			98.47 (3.3)
Number of PWP’s crime & violent/disruptive behaviors, last year			1
Visit to PWP, n(%)			
Frequency: Semi-monthly	1 (3.1)	Methods: Face-to-face only	5 (15.62)
Once every 2 months	6 (18.8)	Telephone only	1 (3.1)
Quarterly	24 (75)	Mixed	26 (81.3)
Half-yearly	1 (3.1)	Full coverage of patients	27 (84.4)
Number of mental-health-related training for village doctors in last year*, n(%)			
0			2 (6.3)
1			12 (37.5)
2–4			12 (37.5)
> 4			5 (15.7)
Top barriers for work implementation, frequencies			
Lacking/insufficient cooperation from patients and/or families (including refusal to physical exams, medication & visits from MHOs, communication with patients and/or families)			29
Bad adherence (to medication) of patients			5
Heavy workload and pressure as being a part-time MHOs			4

*There is one missing data

#### Barriers for implementation

Insufficient and poorly trained human resources, funding without guarantee and low acceptability of service recipients were listed by interviewees and survey respondents as major barriers for implementation.

##### Human resources

The extremely limited human resources made working part-time very common, even within mental healthcare network. MHOs complained of excessively heavy work burden and pressure (Table 6). Lacking professionals also explained for the inactive implementation of non-health departments.

*“… the staff of (Liuyang MHC) are mostly part-time, consisting of leaders, members of the Medical Administration Department and clinicians in our hospital. According to (public mental health) work requirements for this period, our hospital will organize meetings and allocate human resources and funding as needed… Mental health professionals are in extreme short supply. There are not enough mental health professionals for clinical services, let alone for management… There are no (additional) psychiatrists or psychologists for other hospitals to provide psychological consultations” (A, Policy formulator and implementer).*

*“(There are) only two full-time staff, including myself (for Liuyang MHC). We are responsible for supervision and guidance for local (communities/towns) mental health work.”(C, Policy formulator and implementer).*

Poor training of human resources was another concern. No MHOs had education background of mental health. Moreover, quick personal turnovers made the average chance of training less than once per person year (Table 6).

##### Financing

The funding problem was frequently mentioned by policy implementers from different levels. In addition to the reduced funding compared with original budget on Liuyang MHC, there were uncertainty of financing general mental health work and funding competition between mental health and other public health projects. The financial situation was even worse at town and village levels.

*“… next-year government budget (for our hospital, including Liuyang MHC) is based on how much work we have done in this year. If economic growth is good and the government has a larger surplus, we will get a higher budget approved. (Interviewer: Any budget specific for public mental health?) No …” (D, Policy formulator and implementer).*

*“… (MHOs report that) there are financial problems (in their work). Funding for public health is a package; the allocation for public mental health is less.”(C, Policy formulator and implementer).*

##### Acceptability

Un-cooperation of patients and/or their families was listed by MHOs as the top problem in their work.

*“… some patients frequently change their telephone numbers … , reluctant to be contacted by MHOs.” (E, Policy implementer).*

Reasons provided by the front-line implementers and patients’ family members included privacy concern, unsatisfactory effect of medication, no or low family support to patients.

*“… my mom and brother are not willing to take medicine or receive MHO’s management. They think the medication or management is useless (to their disease, referring to schizophrenia) … perhaps, using better medicine will improve the situation (referring to un-cooperation to medication and PWP management and intervention).”(F, Policy recipient).*

## Discussion

In general, Liuyang Policy and Liuyang Plan demonstrate a good example of promoting and implementing China’s national mental health policies at local level. The formulation of Liuyang Policy and Liuyang Plan was an active response to national policies and a pioneering local attempt within Hunan Province and even nationwide [20]. Due to the background of its formulation, the content of Liuyang Policy and Liuyang Plan has strong connections with national mental health policies. In terms of policy implementation, the case of Liuyang shows mutual promotion between national and local policies.

As a snapshot of China’s mental health policy and also a reflection of mental health policy implementation in developing countries, Liuyang presents common problems, lessons and recommendations for wider application:

(1) Mental health should not be restricted to health department; instead, full and wide involvement of stakeholders (particularly patients and their families) and approval by the highest administration within the jurisdiction will strongly enhance the success of mental health policies. The formulation and “semi-formal” approval of Liuyang Policy and Liuyang Plan were completed within the local health system and without policy recipients’ involvement. Therefore, in Liuyang, policy binding for municipal government and non-health departments was extremely limited and the missing of consultation further led to implementation deviations, partial implementation and problems in acceptability. The missing of stakeholders’ involvement and highest-level approval is not only confined to local mental health policies and to China. China’s National Mental Health Plan (2002–2010) is also without approval by ministries of Finance and Education. As a result, China’s National Mental Health Plan (2002–2010) encountered poor enforcement of funding and interventions for children and adolescents [48]. Without full involvement of stakeholders and high-level approval, mental health policies in other developing countries like Ghana, South Africa, Uganda and Zambia also cannot get full implementation, especially at local level [28, 29].

(2) Operational issues, including time frames, targets, evaluation indicators, funding and responsibilities of implementing agencies, should be clearly stated in policy documents. This point is extremely important for policy implementation, especially for local policies. Liuyang Policy had a complimentary plan for implementation and set evaluation indicators for some policy strategies; however, there were still loopholes impeding implementation quality. Another specific example is that no detailed budget in Liuyang Policy and Liuyang Plan makes funding for mental health unguaranteed, and the same problem exists even in the latest National Mental Health Plan (2015–2020) [4]. No accompanying strategic mental health plan is also listed as one barrier for implementation in evaluation studies on African countries’ mental health policies [29].

(3) Clear and narrow policy focuses will be a good choice, especially under resource-constrained situation and for local level. Seriously under-resourced and under-developed mental health systems are commonly found in many countries [49]. In China, insufficient human resources and funding are structural barriers for mental health development and the situation will take a long time to change [45, 50–52]. Local regions, like Liuyang Municipality, have even less mental health resources for utilization. Therefore, though the WHO checklists suggest 12 action areas for mental health policies, it is impossible and unnecessary to equally cover and implement all areas in one local policy. The case of Liuyang demonstrates that identifying priorities according to local situation and concentrating resources on their implementation will gain better effects.

### Limitation

The purpose of this study is to identify problems of Liuyang Policy and Liuyang Plan, and rapid appraisal provides us a useful, quick and low-costs choice. Despite its advantages, rapid appraisal cannot provide as comprehensive information as rigorous evaluations can. In this study, though we have made full use of key-informant interviews, mini surveys and secondary data, there is still some information unavailable, like the prevalence of mental disorders among children and adolescents. This problem can be solved in the end-point evaluation, in which large-scale representative sample surveys covering several themes will be needed.

In semi-structured interviews, a low percentage of respondents were recorded. As one important task for interviews is to understand problems of Liuyang Policy and Liuyang Plan, only making people to talk freely under minimal pressure will gain a fruitful end [31]. Therefore, in semi-structured interviews, when respondents showed reluctant to be recorded, we did not proceed further persuasion. This is the main reason for the low percentage of recording interviews. Though note-taking may miss some details than recording, it is a worthy comprise to obtain stakeholders’ truthful opinions on the policy.

There are also limitations on results of semi-structured interviews and survey among MHOs. First, without correct contact information, we cannot interview PWP and/or their families who are not receiving or refuse psychosis management and intervention. As a result, our interviews only with patient households receiving psychosis management and interventions reveal limited implementation problems from the perspective of policy recipients, especially on their acceptability to Liuyang Policy and Liuyang Plan. Second, as this is an independent third-party evaluation without government mandate, we could not obtain interview permission and data from all related departments, especially the non-health ones. Therefore, the implementation progress of policy strategies, like providing psychological consultation services in primary and middle schools, was found from key informants within the health system. Third, because of voluntary principle, we only received 32 questionnaires in the survey among MHOs. The survey results can only reflect situation of mental health human resources and work in 86.5% of Liuyang’s town hospitals or community healthcare centers.

### Conclusion

This study demonstrates that the case of Liuyang is an active attempt to promote and implement national mental health policies at local level. Its highlights include evidence-based formulation, a complete policy structure, narrow policy priorities under a resource-constrained setting, and satisfactory implementation progress of certain strategies. However, problems also exist, mainly in stakeholders’ involvement, approval, and operational issues. Those problems lead to none or poor implementation of some policy activities. Liuyang’s problems are common in China’s and other developing countries’ mental health policies and its lessons suggested that solid evidence base, high-level approval, full involvement of multiple stakeholders, detailed and comprehensive arrangements in operational issues, and clear policy focuses will promote successful implementation of a mental health policy.

## Additional files

Additional file 1: Appendixes 1 to 5. (DOCX 43 kb)

## Abbreviations

GDP: Gross domestic product
Liuyang Plan: The Plan of Mental Health Work in Liuyang Municipality
Liuyang Policy: The Mid- and Long-term Development Plan for Mental Health in Liuyang Municipality
MHC: Mental Health Prevention and Treatment Center
MHO: Mental health officer
NCMIPP: National Continuing Management and Intervention Program for Psychosis
PH: Psychiatry Hospital
PWP: Patients with psychosis
WHO-AIMS: World Health Organization-Assessment Instrument for Mental Health Systems

## References

[1] Phillips MR, Zhang J, Shi Q (2009). Prevalence, treatment, and associated disability of mental disorders in four provinces in China during 2001-05: an epidemiological survey. Lancet.

[2] Yang G, Wang Y, Zeng Y (2013). Rapid health transition in China, 1990-2010: findings from the global burden of disease study 2010. Lancet.

[3] Ministry of Health, Ministry of Civil Affairs, Ministry of Public Security, et al. The National Mental Health Plan (2002–2010). Shanghai Arch Psychiatry. 2003;15:125-128

[4] Ministry of Health, Office of the Central Committee for Comprehensive Management of Social Security, National Development and Reform Commission, et al. The National Mental Health Plan (2015–2020). 2015. Available from: http://www.nhfpc.gov.cn/jkj/s5888/201506/1e7c77dcfeb4440892b7dfd19fa82bdd.shtml

[5] Ministry of Health, Ministry of Education, Ministry of Public Security, et al. The Proposal on Further Strengthening Mental Health Work. 2004. Available from: http://www.gov.cn/gongbao/content/2004/content_62998.htm

[6] Ministry of Health. The Guidelines for the Development of National Mental Health System (2008–2015). 2008. Available from: http://www.moh.gov.cn/jkj/s5888/200805/81047a30f3c34141b12481de35930d78.shtml

[7] Ministry of Health. Notice on implementing National Continuing Management and intervention program for Psychosis 2006. Available from: http://www.china.com.cn/law/flfg/txt/2006-08/08/content_7056414.htm

[8] Ministry of Health. Specifications for Psychosis Management and Intervention (Version 2009). Available from: http://www.nhfpc.gov.cn/mohbgt/s9514/200911/44384.shtml

[9] Ministry of Health. Specifications for Psychosis Management and Intervention (Version 2012). Available from: http://www.moh.gov.cn/cmsresources/mohgjhzs/cmsrsdocument/doc14594.pdf

[10] National People’s Congress. Mental Health Law of China. 2012. Available from: http://www.moh.gov.cn/zwgkzt/pfl/201301/20969fdf44934b86a0729fb4de33e1ff.shtml

[11] Zhejiang Departments of Health, Finance, Civil Affairs and Public Security. Mental health plan (2002–2010). 2002. Available from: http://www.zj.gov.cn/art/2002/12/24/art_5495_271535.html.

[12] Qingdao Municipal Government. Mental health plan in Qingdao Municipality (2004-2010). 2004. Available from: http://www.qingdao.gov.cn/n172/n68422/n1527/n62802/100020040901478750.html

[13] Government of Guangdong Province. Mental health plan in Guangdong Province (2016–2020). 2016. Available from: http://www.zhsi.gov.cn/zhsi_news/subpage_sb.jsp?id=26217

[14] Government of Yinchuan Municipality. Mental health plan in Yinchuan Municipality. 2018. Available from: http://www.yinchuan.gov.cn/xxgk/bmxxgkml/szfbgt/xxgkml_1841/zfwj/yzbf/201802/t20180214_691797.html.

[15] Jansson E, Fosse E, Tillgren P (2011). National public health policy in a local context--implementation in two Swedish municipalities. Health Policy.

[16] Saito J, Keosada N, Tomokawa S (2015). Factors influencing the National School Health Policy implementation in Lao PDR: a multi-level case study. Health Promot Int.

[17] Draper CE, Lund C, Kleintjes S (2009). Mental health policy in South Africa: development process and content. Health Policy Plan.

[18] Di XK, Xiao SY (2012). Assessment of contents of local mental health laws in mainland China. Chin Ment Health J.

[19] WHO. WHO-AIMS Report on mental health system in Hunan province of the People's Republic of China. WHO: World Health Organization. 2006. Available from: https://www.who.int/mental_health/china_hunan_who_aims_report.pdf

[20] Song DM, He JY, Zhou WJ (2011). Primary development of community mental health service in Liuyang. Chin Ment Health J.

[21] Liuyang Municipal Government. Statistical Communiqué of Liuyang municipality on the 2015 economic and Soc Dev 2016. Available from: http://www.liuyang.gov.cn/liuyanggov/sjkf/njgb/3851611/

[22] Provincial Bureau of Statistics of Hunan. Statistical Communiqué of Hunan Province on the 2015 economic and. Soc Dev. 2016; Available from: http://www.hntj.gov.cn/tjfx/tjgb/jjfzgb/201603/t20160317_4326853.html.

[23] National Bureau of Statistics of China. Statistical Communiqué of the People’s republic of China on the 2015 National Economic and. Soc Dev. 2016; Available from: http://www.stats.gov.cn/tjsj/zxfb/201602/t20160229_1323991.html.

[24] National Health and Family Planning Commission (2016). China health and family planning statistics yearbook.

[25] WHO. Monitoring and evaluation of mental health policies and plans. World Health Organization. 2007. Available from: http://www.who.int/mental_health/policy/services/14-monitoring%20evaluation_HKprinter.pdf

[26] Funk M, Freeman M (2011). Framework and methodology for evaluating mental health policy and plans. Int J Health Plann Manag.

[27] World Health Organization. World Health Organization Assessment Instrument for Mental Health Systems (AIMS) Version 2.2. 2005. World Health Organization. Available from: http://www.who.int/mental_health/evidence/AIMS_WHO_2_2.pdf?ua=1

[28] Awenva AD, Read UM, Ofori-Attah AL (2010). From mental health policy development in Ghana to implementation: what are the barriers?. African J Psych.

[29] Faydi E, Funk M, Kleintjes S (2011). An assessment of mental health policy in Ghana, South Africa, Uganda and Zambia. Health Res Policy Systs.

[30] Kleintjes S, Lund C, Swartz L (2010). Mental health care user participation in mental health policy development and implementation in South Africa. Int Rev Psychiatry.

[31] Beebe J (1995). Basic concepts and techniques of rapid appraisal. Hum Organ.

[32] Trotter RT, Needle RH, Goosby E (2001). A methodological model for rapid assessment, response, and evaluation: the RARE program in public health. Field Methods.

[33] Murray SA (1999). Experiences with "rapid appraisal" in primary care: involving the public in assessing health needs, orientating staff, and educating medical students. BMJ.

[34] Besada D, Rohde S, Goga A (2016). Strategies to improve male involvement in PMTCT option B+ in four African countries: a qualitative rapid appraisal. Glob Health Action.

[35] Fusch PI, Ness LR (2015). Are we there yet? Data Saturation in Qualitative Research. Qual Rep.

[36] Smith J, Firth J (2011). Qualitative data analysis: the framework approach. Nurse Res.

[37] Gale NK, Heath G, Cameron E (2013). Using the framework method for the analysis of qualitative data in multi-disciplinary health research. BMC Med Res Methodol.

[38] Liuyang Municipal Government. The Twelfth Five-Year Plan of Liuyang Regional Health Development (2011-2015). 2011. Available from: http://www.changsha.gov.cn/xxgk/qsxxxgkml/lys/ghjh_4806/zzqgh_4807/201207/t20120713_345323.html

[39] Zhang QW. Mental health services utilization and related factors among patients with schizophrenia in rural communities: Ph.D. dissertation of Central South University; 2008.

[40] Gui LH. Epidemiologic study on depression among rural residents in Liuyang: Ph.D. dissertation of Central South University; 2009.

[41] State Council (2006). The approval of establishing the inter-ministerial joint conference system for mental health work.

[42] Yu Y, Liu ZW, Hu M (2015). Assessment of mental health literacy using a multifaceted measure among a Chinese rural population. BMJ Open.

[43] Liu F. Knowledge of and attitude toward mental health illnesses of health workers in township health centers of Liuyang: Master dissertation of Central South University; 2010.

[44] Ministry of Health. The Evaluation Criteria for Psychosis Management and Intervention. 2012. Available from: http://www.gov.cn/gzdt/2012-08/06/content_2199291.htm

[45] Liu J, Ma H, He YL (2011). Mental health system in China: history, recent service reform and future challenges. World Psychiatry.

[46] Zhou W, Xiao SY (2014). Current mental health policies outside China. Chin Ment Health J.

[47] Wang X, Ma N, Wang LY (2016). Management and services for psychosis in People’s republic of China in 2014. Chin Ment Health J.

[48] Ma H, Liu J, Yu X (2009). The development and interpretations of China’s key mental health policies of the recent decade. Chin Ment Health J.

[49] Knapp M, Funk M, Curran C (2006). Economic barriers to better mental health practice and policy. Health Policy Plan.

[50] Su K, Sun X, Zhang Y (2012). Mental health services in China: a review of delivery and policy issues in 1949-2009. Chin Ment Health J.

[51] Wong DFK, Zhuang XY, Pan JY (2014). A critical review of mental health and mental health-related policies in China: more actions required. Int J Soc Welf.

[52] Zhao DY (2012). The incremental logic of China mental health policy making: an interpretation toward the road of mental health legislation. Chin J Health Policy.

