# Supplementary material for: Evaluating China’s mental health policy on local-level promotion and implementation: a case study of Liuyang Municipality
Source: BMC Public Health. 2019 Jan 7;19:24. doi: 10.1186/s12889-018-6315-7 (PMC6323835; doi:10.1186/s12889-018-6315-7)
Supplement: Supplementary file 1 — Appendixes 1 to 5. (DOCX 43 kb) [file 12889_2018_6315_MOESM1_ESM.docx]

**Appendixes**

**Appendix 1 Translated full texts of Liuyang Policy**

**Medial and Long-term Development Plan for Mental Health in Liuyang City**

In order to accelerate the development of the mental health system in our city, strengthen the leadership and management of mental health tasks, guarantee the benefits of mental health patients, improve the mental health level of the general population, insure the steadiness of our city, reduce the phenomena of “entering poverty because of diseases” and “re-entering poverty because of diseases”, and establish a harmonious society of socialism, we have designed the medial and long-term development plan for mental health in Liuyang city on the basis of Liuyang’s actual current situation.

**1. Current Situation**

There are 1,340,000 people in our city, and it is estimated that 20,000 have psychiatric disorders, less than 30% of whom have sought treatment before and less than 10% who have received regular treatment. Additionally, the troubles and accidents involving psychiatric patients happen frequently, the rate of severe psychiatric disorders is rising, and the rate of mental health literacy is less than 30%. There are thousands of adolescents suffering from emotional and behavioral problems. Alzheimer's disease, depression, and drug abuse are becoming increasingly severe.

**2. Guiding Principles**

2.1 To achieve the tenet of “mental health for all”, promote mental health services to fulfil the public’s demands, and adapt to the social and financial development in our city, under the guidance of Deng Xiaoping Theories, Secretary General Jiang's "Three Represents Theory", and Secretary General Hu's “Building Harmonious Society of Socialism”.

2.2 Comply with the working principle “primarily prevent, integrate of prevention and treatment, intervene according to severity of the problem, cover broadly, and manage legally” to advance the development of mental health tasks across our city.

**3. Objectives**

3.1 Establish the mental health system of multi-department cooperation and the societies’ participation, and mechanism of management and coordination.

3.2 Strengthen the publicizing of mental health literacy and health education, improve the whole society’s understanding of the significance of mental health work, and enhance the mental health level of the general population. The rate of mental health literacy should be increased to 80%.

3.3 Enhance the medical treatment and rehabilitation services for people with severe psychiatric disorders, avoid the increasing burden of mental health diseases effectively, prevent the occurrence of the phenomena “entering poverty because of diseases” and “re-entering poverty because of diseases”, relief the social labour force , and decrease the effects of disabilities of psychiatric disorders.

3.4 Set up an integrated mental health services system and network, consummate the function of current mental health institutions, increase staff quality and treatment expertise in the mental health delivery system, and meet the public’s demand for basic mental health services.

**4. Organization and Management**

4.1 The leadership team is operated by the city government, whose staff members come from the agencies such as Disabled People’s Federation. The leadership team is in charge of supervising all mental health tasks in the city, integrating the mental health project with the City’s financial budget and social development plan, and putting them into effect.

4.2 Set up the department coordination system

Every department or agency, such as Health, Education, Public Security, Civil Affairs, Finance, Disabled People’s Association, Justice and Labor, should focus on the increasingly severe mental health problems, by adapting effective prevention and control measures for their own responsibilities, both separately and in cooperation.

**5. Measures**

5.1 Consummate the construction of a three-level network

5.1.1 Liuyang Mental Health Prevention and Treatment Center, which operates in conjunction with Liuyang Mental Health Hospital, would be set up by the efforts of the City Health Bureau. The Mental Health Prevention and Treatment Center has two offices, psychiatric health and psychological health, which include five work teams: the Medical team, Technical team, Health Education team, Information Network team, and Guide team, to be responsible and in charge of the implementation of psychiatric and psychological work in our city. The three-level prevention, treatment and rehabilitation network, which includes Liuyang Mental Health Prevention and Treatment Center, township mental health prevention and treatment office, and village clinic, neighbourhood committee and family members supervision sites.

The missions and responsibilities of the Mental Health Prevention and Treatment Center in Liuyang City are the following:

① Generalize and disseminate mental health knowledge through multiple approaches and through multiple channels

② Take charge of providing training and technological guidance to the mental health professionals in every town

③ Take responsibility for examination and risk assessment to determine and adjust the treatment schemes and measures in every town over a certain period (every 2 months)

④ Establish Liuyang mental health information website, and the economic database of psychiatric patients in Liuyang City; meanwhile, control any change or development

⑤ Take charge of organizing and coordinating with the tasks on education, training and evaluation of psychological consultation, and expand the work of psychological consultation and examination

⑥ Take the responsibility for assessing the implementation of mental health tasks in the city

5.1.2 Establish a public health office in every township hospital in which there are one to two full-time or part-time mental health experts. The responsibilities of the experts are:

① Establish the information cards and databases of psychiatric patients in the precinct such as electronic archive, and enter the information into the database

② Follow up and supervise the psychiatric patients in the precinct

③ Take the responsibility for screening psychiatric patients in the precinct

④ Take the responsibility for primary training on mental health literacy to psychiatric patients and their families in the precinct

⑤ Take the responsibility for psychological examination work

5.1.3 The responsibility of village health centers, village committees, and family members:

① Take the responsibility for the overall supervision of psychiatric patients

② Prevent psychiatric patients’ impulsive behaviours or behaviours that may produce harm to the society, and ensure they receive timely hospital involvement

③ Develop a mechanism to quickly report a positive psychiatric screen or assessment

5.1.4 Staffing quotas

①There are 30 persons in the Mental Health Prevention and Treatment Center in Liuyang City, that include: 2 administrative staff, 10 on the medical team, 5 on the technical team, 5 on the health education team, 4 on the information network team, and 4 on the guide team.

②There are one to two full-time or part-time mental health experts in every town’s public health office.

5.2 Strengthen operation training, improve staff ability

The Mental Health Prevention and Treatment Center will give mental health experts regular operation training and circle training, in order to improve the staff’s professional ability, ensure the steadiness and consistency of teams’ staff, and improve the quality of services. Psychological consultation services should be deeply and broadly implemented in multiple ways and through multiple channels to resolve the mental health problems for those who seek help.

5.3 Enhance the information management of psychiatric patients.

It is necessary to equip the Mental Health Prevention and Treatment Center with a computer platform system. The public health offices in every town should record the basic information and development information of psychiatric patients immediately and correctly. Therefore, the Mental Health Prevention and Treatment Center could give evidence for policy making and measures that would be taken by controlling the information regarding all psychiatric patients through the website.

5.4 Strengthen the medical treatment and rehabilitation work for patients with severe psychosis.

5.4.1 The medical treatment and rehabilitation work for patients with severe psychosis is critical for achieving “mental health for all”. The city psychiatric hospital should take positive measures to apply the appropriate medical and rehabilitative guarantee to patients with severe psychosis.

5.4.2 Improve the Detection Treatment rates. The goal is that the screening rate of psychiatric diseases should be more than 80% in 2015. In addition, the treatment rate of schizophrenia should be more than 60%, guardianship rate should be more than 95%, and the rate of significant improvement should be more than 70%. We expect to have nearly eliminated violent events or trouble caused by psychiatric patients, which contribute to the steadiness of society.

5.4.3 Strengthen the supervision to “three-no-people”, poor persons without jobs, and poor rural persons among psychiatric patients; and supply the medical aid and relief to psychiatric patients, who are arrested, through the multiple channels of government, society, and family.

5.4.4 Enhance interventions for psycho-behavioral problems among the key population and decrease the occurrences of psychiatric diseases.

① Set up the psychological consultation and education clinics in city-level general hospitals and maternal and children’s hospitals, conduct mental health knowledge training, and prevent psychiatric disorders when they are at the primary stage.

② Middle schools and elementary schools should set up mental health and quality education and the training of adaption ability to improve the children and adolescents’ adaption ability and ability to deal with emergencies.

③ Develop the dissemination of elders’ mental health, publicize the prevention knowledge of psychiatric disorders, such as Alzheimer’s disease and depression; take psychological consultation activities by focusing on the elders’ characteristics and the problems faced; and then supply the effective support and help to improve elders’ life quality.

5.5 Financial Foundation

The city financial budget should include the mental health system, and ensure that the financial support is on track, then guarantee that mental health tasks can be implemented smoothly.

**Appendix 2 Translated full texts of Liuyang Plan**

**Plan of Mental Health Work in Liuyang City**

Psychiatric disorder is one category of the most serious diseases that harm people’s physical and mental health. Along with the rapid development of social economy, the increase in various conflicts and the stress of competition, the incidence of psychiatric disorders appears to be rising obviously. There are now 1,340,000 people in our city and according to the study results of the “Public Health Comprehensive Trials”, there are approximately 20,000 individuals with severe psychiatric disorders patients in our city. Mental health is becoming the most significant public health and social problem. In order to strengthen our approach to implementing mental health work, develop excellence in mental health treatment and prevention, reduce and prevent the occurrences of various psycho-behavioral problems, enhance the physical and mental health of the overall population, and promote ongoing economic and social development, we have designed an implementation plan based on regulation “The announcement of opinions on strengthen mental health work by the Department of Health” by People's Government Office Department of Hunan Province (Xiang Government NO.[2005]34), and the actual situation in our city.

**1. Guiding Principles**

Mental health work should occur according to the following principles: “primarily prevent, integrate of prevention and treatment, intervene according to severity of the problem, cover broadly, and manage legally”, and under the system: “the government leads, departments cooperate, and society participates” in order to advance the development of mental health work. We must also establish a mental health services network, and the key point of prevention and treatment work should be switched to communities and fundamental organizations. A multi-channel model to raise capital must be established, whose main resource is from government and whose main purpose is to guarantee the implementation of the prevention and treatment of psychiatric disorders. We should also strengthen the treatment and rehabilitation of severe psychotic disorders, focus on the interventions to the psychological behavioural problems among key populations, provide treatment and rescue to psychiatric patients, improve the public population’s self-protection consciousness, fulfil the public’s demands to mental health services to the greatest extent, establish and consummate the policies and regulations of mental health tasks, and enhance the mental health work team construction as well as their scientific research work.

**2. Goals**

According to the “China Mental Health Development Plan” by the [Ministry of Health](http://www.health.go.ke/), Ministry of Civil Affairs, [Ministry of Public Security, and](http://blog.tradeknow.com/simple/index-htm-p-38737-type-blog.html) China Disabled People’s Federation, “The announcement of opinions on strengthen mental health work by the Department of Health” by People's Government Office Department of Hunan Province, and the actual situation in the city, our mental health task goals during the Eleventh Plan are: to achieve the rate of prevention literacy among the general population regarding mental health and psychiatric disorders of 50%; decrease the incidence of psychiatric disorders and psycho-behavioural problems in children and adolescent by 12%; increase the rate of schizophrenia patients receiving treatments to 60%; increase the coverage rate of mental health prevention, treatment, and rehabilitation work to 75%; the rate of emergencies or problems caused by people with psychiatric disorders should be decreased under 0.2%; the rate of psychiatric patients under surveillances should be increased to 90%; and the amount of professional staff members trained rate should be 80%.

**3. Measures**

3.1 Establish the cooperation system led by the government. We have to set up the mental health leadership team in which people from city government is the team leader and the staff members are leaders from departments of Health, Education, Public Security, Justice, Civil Affairs, Finance, Disabled People’s Federation, and any other societies; and form the organizational and management systems which are led by government, works in cooperation with related agencies, and involves the entire society. We should also involve the mental health work into the ongoing economic and social development plan in our city to ensure the achievement of mental health work goals.

3.2 Establish and integrate the mental health services system and network

3.2.1 Set up the Liuyang Mental Health Prevention and Treatment Center which is based in Liuyang Mental Health Hospital. There are four teams: 1. Technical team, which is in charge of examination and treatment of psychiatric disorders, treatment of patients with severe psychiatric illness, providing professional training to mental health staff members across organizations and community services; 2. Health Education team, which is in the charge of the publicity of health knowledge as well as health knowledge education training to patients and their families; 3. Information Network team, which is in charge of establishing the surveillance system of psychiatric disorders in the city and recording and supervising the information on psychiatric disorders; 4. Emergency Intervention team, which is in charge of dealing with public health emergencies. All of the upper county level general hospitals should set up psychiatric clinics, which should include 1-2 professional technical members. Every town should also set up mental health offices which has at least one professional technical mental health expert. Therefore, we will form the mental health services system and network which is driven by the Liuyang Mental Health Prevention and Treatment Center, and supported by psychiatric departments in general hospitals, the local medical care organizations, and psychiatric social rehabilitation organizations.

3.2.2 Staffing Quotas and Financial Budget

① The staffing quota in Liuyang Mental Health Prevention and Treatment Center is 30, which includes 2 administration members, 15 on the technical team, 5 on the health education team, 4 on the information network team, 4 on the guide team, and 4 on the emergency intervention team. The financial budget is counted by 30,000 Yuan per year per person:

30 Persons×30,000 Yuan /Year＝900,000Yuan

② Work Budget: includes instruments, official materials, fees for conferences and training, will require approximately 300,000Yuan.Therefore, the total financial budget is approximately 1,200,000 Yuan.

3.3 Establish the mechanisms by which mental health work implementation is guaranteed by the government

3.3.1 Financial guarantee. Expand the financial support of mental health work, integrate the mental health work into the government financial budget, develop the entire budget, ensure sufficient funding reaches mental health organizations and mental health workers, and guarantee the successful implementation of mental health work.

3.3.2 Policy guarantee. We will establish the allowance policy which is good for mental health problems prevention and treatment work, and accelerates the mental health legislation process to supply the law and policy guarantee to mental health work.

3.4 Strengthen the propaganda and popularization of mental health knowledge. We should conduct the mental health knowledge propaganda positively, and publicize the mental health knowledge to the general population by television, newspaper, radio, brochures, and folk culture activities around every year’s “World Mental Health Day” on October 10^th^, to improve the mental health level of the general population, and eliminate the discrimination of psychiatric patients.

3.5 Strengthen the psycho-behavioural intervention to target populations

3.5.1 Focus on prevention and intervention approaches to children and adolescents’ psycho-behavioural problems. The departments of Health and Education should work together on the mental health education and knowledge training of teachers and health providers in the schools, in order to improve the ability to discover the children and adolescents’ psycho-behavioral problems. The education departments must integrate mental health education into the teaching schedule; conduct testing of children and adolescents’ psychological and behavioural status based on the existing team and network in the schools and under the guidance of professional members from the Mental Health Prevention and Treatment Center; and provide mental health education (including skills training) and consultation services by focusing on their characteristics.

3.5.2 Enhance research and interventions on women’s psycho-behavioural problems and psychiatric disorders. Women’s Federation and Maternal and Children’s Health Care Center should strengthen mental health care for women during the pregnancy and perinatal and climactenic periods; enhance the identification and handling of common psychological problems; decrease the incidence of negative psychological reactions during prenatal, postnatal and climactenic period; carry out the mental health consultation and crisis intervention services to rural women; and adapt effective measures to decrease the prevalence of psychiatric disorders among rural women.

3.5.3 Carry out the mental health propaganda and psychiatric disorders interventions to elders. We could establish the intervention network for Alzheimer’s disease by utilizing existing mental health resources. All levels of the elders’ associations should adopt effective measures according to local conditions, conduct the elders’ mental health propaganda among senior people and their family members and caregivers, publicize the prevention knowledge of geriatric psychiatric disorders such as Alzheimer’s disease and depression and common health problems, carry out the mental health consultation to supply effective support and help, and improve senior people’s quality of life.

3.6 Accelerate the development process of the mental health work team.

Strengthen the training and development of human resources which address mental health. We should establish on-the-job training to existing mental health professionals and training on mental health knowledge to other medical staff members and other workers who engage in mental health work. We should also enhance the ability for early-stage identification and effective handling to psychiatric disorders. We will enhance the occupation morality, occupation discipline and medical ethics education of mental health services providers and reinforce the legal concepts and services consciousness. Finally, we must take measures to improve the working conditions and quality of life for mental health services providers, and promote the development of mental the health work team.

**Appendix 3 The list of documentation reviewed**

**Category One: National and local policies**

1. The National Mental Health Plan (2002-2010). 2002.
2. The Proposal on Further Strengthening Mental Health Work. 2004.
3. Notice on implementing National Continuing Management and Intervention Program for Psychosis. 2006.
4. The Approval of Establishing the Inter-ministerial Joint Conference System for Mental Health Work. 2006.
5. The Approval of Enlarging Members of the Inter-ministerial Joint Conference System for Mental Health Work. 2007.
6. The Guidelines for the Development of National Mental Health System (2008–2015). 2008.
7. Specifications for Psychosis Management and Intervention (Version 2009). 2009
8. The Proposal on Promoting Equal Access to Basic Public Health Services.2009
9. National Plan on Constructing and Developing Prevention and Treatment System of Mental Health. 2010.
10. Mental Health Law of China. 2012.
11. Specifications for Psychosis Management and Intervention (Version 2012). 2012
12. The Evaluation Criteria for Psychosis Management and Intervention. 2012.
13. Measures for the Management of Psychoses Reporting (Trial). 2013.
14. Notification on Promoting to Open Psychiatric Outpatient in General Hospital. 2013.
15. The National Mental Health Plan (2015-2020).2015.
16. Government Illustrations on The National Mental Health Plan (2015-2020).2015.
17. The Twelfth Five-Year Plan of Liuyang Regional Health Development (2011-2015). 2011.

**Category Two: Government reports**

1. Statistical Communiqué of Hunan Province on the 2015 Economic and Social Development. 2016.
2. Statistical Communiqué of the People’s Republic of China on the 2015 National Economic and Social Development. 2016.
3. Liuyang Government Work Report. 2015.
4. General Information of Liuyang. 2015.

**Category Three: Researches**

1. Zhang QW. Mental health services utilization and related factors among patients with schizophrenia in rural communities. Ph.D. dissertation of Central South University, 2008
2. He XY. Depression symptoms in Liuyang rural community-dwelling elderly: prevalence and associated factors. Master dissertation of Central South University, 2008
3. Gui LH. Epidemiologic study on depression among rural residents in Liuyang. Ph.D. dissertation of Central South University, 2009
4. Gui LH, Xiao SY. Prevalence and related factors of suicidal ideation and attempts in patients with depressive episode in rural community in Liuyang City. Chinese Mental Health Journal. 2009; 23: 651-655
5. Zhang QW, Xiao SY, Zhou L, et al. Treatment status and related factors among patients with schizophrenia in a rural Chinese community. Chinese Mental Health Journal. 2010; 24: 241-249
6. Zhang DX. Recognition and treatment of depression in township health centers of Liuyang: a participant observation study. Master dissertation of Central South University, 2010
7. Liu F. Knowledge of and attitude toward mental health illnesses of health workers in township health centers of Liuyang. Master dissertation of Central South University, 2010
8. Song DM, He JY, Zhou WJ, et al: Primary development of community mental health service in Liuyang, Chinese Mental Health Journal. 2011; 25: 517-520
9. Luo JJ. Willingness to seek professional help for psychological problems and associated factors among rural residents in Liuyang County. Master dissertation of Central South University, 2012
10. Hu M. Social ties, social support and emotional distress among elderly in rural area. Ph.D. dissertation of Central South University, 2012
11. Yu Y, Liu ZW, Hu M, et al. Assessment of mental health literacy using a multifaceted measure among a Chinese rural population, BMJ Open. 2015; 5: e009054

**Category Four: Routine surveillance data**

Quarterly reports on psychosis management and intervention in Liuyang

**Appendix 4 Semi-interview structures**

Section One: Information of Liuyang Policy and Plan

**(For senior leadership)**

1. What was the original intention for formulating Liuyang Policy and Plan? What problem did the policy aim to solve?
2. When and which government department/organization proposed the initial intention for the policy formulation? After that, which department/organization/leader mandated its formulation?
3. During the formulation process, what activities (e.g. stakeholders’ consultation meetings, survey, and documentation review) carried out?
   1. Further questions on stakeholders’ consultation meetings
4. Who participated in the meetings? (e.g. leaders and staff of government departments and hospitals, patients and their families, social societies)
5. Did representatives of each group speak at the meetings? Was consensus reached finally?
6. How many consultation meetings were held?
   1. What information did survey collect? (e.g. prevalence, service resources, mental health need)
   2. What documents were utilized? (e.g. national/regional policies, secondary data, government/research reports, expert opinions)?
7. When did Liuyang Policy and Plan get approval and go into effect? Which department approved and disseminated the two documents?

Section Two: Activity implementation by Liuyang Municipal Government

**(For senior leadership)**

1. Is the leadership team for mental health established according to the requirements stated in Liuyang Policy and Plan? What work (e.g. meetings) does the team carry out?
2. Is the working mechanism of multi-department coordination formed in practice? What forms does the mechanism take of (e.g. working organization, routine meetings)?
3. Does Liuyang Municipal Government set a budget for mental health?

Section Three: Overall situation of the mental health services network

**(For senior leadership)**

1. When did Liuyang MHC start its operation? How many department and staff are there in Liuyang MHC?
2. How much government funding is allocated to MHC? How much is for personnel and daily operation respectively?
3. How many towns/urban communities establish mental health office? How many mental health officers are equipped to each town/urban community?

Section Four: Activity implementation by the mental health services network

**(For senior leadership and staff of Liuyang MHC)**

1. How is the work of mental health education carried out at city, town and village levels?

Detailed information for collection includes: whether conducted, in which forms, work frequencies, targeted populations.

1. How is the Liuyang MHC’s work of mental health professionals’ training carried out?

Detailed information for collection includes: whether conducted, in which forms, work frequencies, whether evaluated effect.

1. How is Liuyang MHC’s work of regular guidance for treatment scheme and risk assessment of psychiatric patients carried out?

Detailed information for collection includes: whether conducted, in which forms, work frequencies, coverage of towns/urban communities.

1. How is the operation of Liuyang mental health information website?

Detailed information for collection includes: whether constructed, whether in operation, website link.

1. How is the work of reporting and recording psychiatric patients in information system carried out at city, town and village levels?

Detailed information for collection includes: whether reported and recorded, relationship with the 686 program, recording rate of psychiatric patients, information updating.

1. How is Liuyang MHC’s evaluation on Liuyang mental health work carried out?

Detailed information for collection includes: whether conducted, in which forms, evaluation frequencies and coverage, evaluation criteria.

1. Apart from traditional medical treatment, does Liuyang Psychiatric Hospital conduct rehabilitation services?
2. What are the implementation barriers for the above activities at city, town and village levels?

**(For mental health officers)**

1. How many mental health officers were there at your town hospitals/community healthcare center? Are you full-time or part-time officers for mental health work?
2. As a mental health officer, what tasks related to mental health promotion and prevention you have been carrying out? How is the implementation of each work?
3. What are the implementation barriers during your work?

Section Five: Activity implementation by organizations outside the mental health services network **(For senior leadership, staff of Liuyang MHC, and mental health officers)**

1. How many and what proportion of general hospitals and maternal & children’s hospitals provide services of mental health education and/or psychological consultation and/or early screening of mental disorders?
2. How many and what proportion of primary & middle schools include mental health into their teaching curriculums and/or provide psychological consultation and/or early screening of mental disorders? How is the work of training teachers and school doctors on mental health?
3. How is the Women’s & Elders’ Federations working on mental health education and early screening of mental disorders?
4. What are the implementation barriers for the above activities?

Section Six: Client experience of mental health services

**(For psychiatric patients and/or families)**

1. Have you been provided with mental health services (e.g. training/ lecture on mental health) by village doctors and/or town hospitals/community healthcare centers and/or Liuyang MHC/Liuyang Psychiatric Hospital and/or other organizations?
2. Have you been routinely visited by village doctors/ mental health officers and been offered professional guidance on your/your family member’s mental disorder? How often?

**Appendix 5 Questionnaire of Liuyang mental health officers**

1. Age: 2. Sex: 3. When did you take the job as a mental health officer:

4. Are you a full-time or part-time mental health officer?

If working part-timely, apart from task related to mental health, what other tasks do you have to take? (e.g. chronic disease management, epidemic disease management, maternal and children healthcare, clinical work, administrative work)

5. The town you are working for has: (If unknown, please fill with “unknown”)

(1) the number of the current permanent resident population

(2) the number of registered patients with psychosis (PWP)

(3) the number of PWP under treatment

(4) the number of crimes and/or violent/disruptive incidents by PWP in the last year

(5) by your last visit, the number of PWP in stable condition

(6) the number of PWP without guardian

6. The major of your higher education is

7. The professional qualification you have is (e.g. Clinical/public health practitioner qualification)

8. How many times of in-service training related to mental health have you received in the past three years?

9. Last year, on which group of population did your town hospital/community healthcare center conduct mental health education? (multiple choices)

(1) PWP; (2) family members of PWP; (3) school students; (4) maternal women;

(5) the elderly; (6) others, please list

10. Last year, what kind of activities was conducted for mental health education? (multiple choices)

(1) lecture; (2) bulletin boards; (3) education through daily contact with PWP and/or their families; (4) others, please list

11. How often do you visit PWP?

The forms of your visit include: (e.g. home visits, telephone interview)

Do you visit all PWP or only visit part of PWP by selection?

If visiting selectively, what is the coverage rate of PWP?

What is your selection standard?

12. Last year, how many times of mental-health-related training were provided by your town hospital to village doctors?

13. Apart from mental health education, PWP visits and training for village doctors, what other tasks related to mental health are conducted by your town hospital?

14. Please list the top three barriers for your work implementation.

15. Are your clients (e.g. patients and their families) cooperative with you work?

If no, please list the main reasons.
